# Presenting a New Standard Drug Model for Turmeric and Its Prized Extract, Curcumin

**DOI:** 10.1155/2018/5023429

**Published:** 2018-01-15

**Authors:** Franco Cavaleri

**Affiliations:** Biologic Pharmamedical Research, 688-2397 King George Blvd., White Rock, BC, Canada V4A7E9

## Abstract

Various parts of the turmeric plant have been used as medicinal treatment for various conditions from ulcers and arthritis to cardiovascular disease and neuroinflammation. The rhizome's curcumin extract is the most studied active constituent, which exhibits an expansive polypharmacology with influence on many key inflammatory markers. Despite the expansive reports of curcucmin's therapeutic value, clinical reliability and research repeatability with curcumin treatment are still poor. The pharmacology must be better understood and reliably mapped if curcumin is to be accepted and used in modern medical applications. Although the polypharmacology of this extract has been considered, in mainstream medicine, to be a drawback, a perspective change reveals a comprehensive and even synergistic shaping of the NF-kB pathway, including transactivation. Much of the inconsistent research data and unreliable clinical outcomes may be due to a lack of standardization which also pervades research standard samples. The possibility of other well-known curcumin by-products contributing in the polypharmacology is also discussed. A new flowchart of crosstalk in transduction pathways that lead to shaping of nuclear NF-kB transactivation is generated and a new calibration or standardization protocol for the extract is proposed which could lead to more consistent data extraction and improved reliability in therapy.

## 1. Introduction

Turmeric is a spice used for thousands of years in Indo-Asian culinary recipes, a significant component of most curry powders [1, 2]. The bright orange-yellow powder that is derived from the boiled, dried, and crushed turmeric rhizome is also used as a potent natural dye and food colouring agent even today [3, 4]. Various parts of the turmeric plant have been used as medicinal treatment for various conditions from ulcers [5] and arthritis [6] to cardiovascular disease [7] and neuroinflammation [8]. Turmeric plays a central role in Ayurveda and other traditional medicines [5, 9–11]. The rhizome's naturally occurring curcuminoid analogues are likely the most studied active constituents [12]; however, the perennial plant contains many other active constituents including a water-soluble peptide, turmerin, and essential oils including turmerones and zingiberene that can contribute pharmacology of their own [13–16].

**Figure 1:**
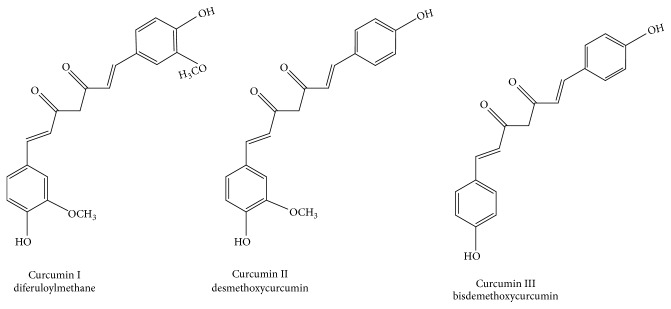
Comparing the structure of the three curcuminoids.

**Figure 2:**
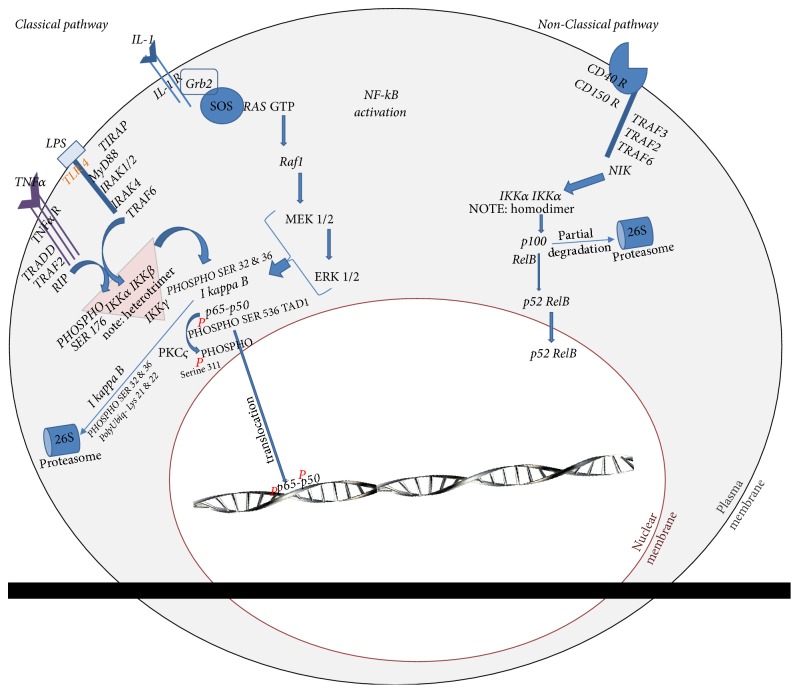
Depiction of classical and nonclassical pathway to NF-kB (p65-p50 or RelBp52) translocation activation. Figure: schematic by Franco Cavaleri.

**Figure 3:**
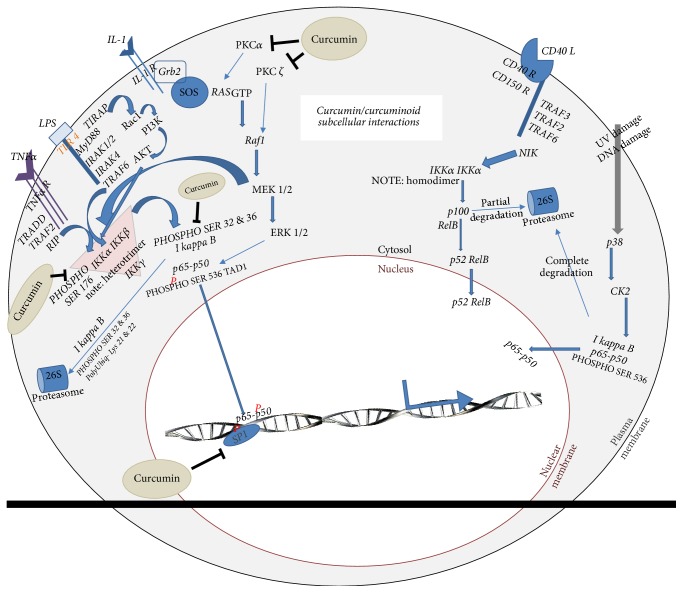
Curcumin polypharmacology plotted to show a rendered influence on p65-p50 transactivation potential showing a relatively more selective outcome if NF-kB is selected as the ultimate target of the polypharmacology.

The curcumin extract, although relatively isolated from the rest of the plant's constituents, still exhibits an expansive polypharmacology [17, 18]. The extract is made up of three main curcuminoid analogues: diferuloylmethane (curcumin I), desmethoxycurcumin (curcumin II), and bisdesmethoxycurcumin (curcumin III) [19]. Each curcuminoid analogue is similar in structure as shown in Figure 1. As we see displayed in Figures 2 and 3, the curcumin extract with its three naturally occurring curcumin analogues targets multiple subcellular proteins in a broad manner [20–22]. This polypharmacology may be a function of the nonspecific activity by each curcuminoid analogue on different targets, but it may also be a function of other factors that will be discussed.

Curcumin has been shown to influence many key biological markers of inflammation such as NF-kB [23, 24] and C-reactive protein [25]; growth factors and growth factor receptors [26]; eicosanoid enzymes such as cyclooxygenase (COX) inhibition [27]; tumor suppressor proteins such as p53 [28]; lipoxygenase (LOX) inhibition [29]; and inhibition of BACE1 and *β*-amyloid aggregation to potentially deliver benefits in Alzheimer's treatment [30–32]. Curcumin modulates various cytokines such as inhibition of interleukins 1, 2, 6, 8, and 12, TNF*α*, and IFN*γ* [24, 33, 34], while inducing the anti-inflammatory IL-10 [35, 36]. Curcumin is also shown to inhibit expression of CD80, CD86, and MHC II in T-cells [33].

However, reliability and research repeatability with this therapeutic agent are still falling short [37]. This may be due to the fact that the initiative has not been taken to establish a standardization protocol for the natural medicinal agent that will set in motion consistent specifications for the curcumin research standard samples being studied. Other factors contributing to the pharmacology such as the well-known curcumin degradation by-products [38, 39] are also likely playing a role.

In this review, curcumin's pharmacology will be discussed in the context of NF-kB-related proteins and their signalling pathways and other subcellular pathways that the extract successfully targets. A new calibration or standardization protocol is proposed with hopes that it may help set the stage for more consistent data extraction and improved reliability in therapy. This correction may facilitate health care professionals' trust in the treatment of inflammatory disorders with curcumin-based therapies.

## 2. Inflammation and the NF-kB Signalling Pathway

Inflammation is a broad term used to describe a complex process by which the body recruits immune system and other biochemicals to eliminate pathogens, autoreactive self-cells and dead cells, and start in motion a restorative and recovery process. Inflammation is characterised by swelling, heat, and pain [40] as we see with acute conditions such as injury. However, the subcellular events associated with inflammation are quite complex and are more recently associated with chronic systemic illnesses that at first thought appear to be far removed from the inflammatory process [41] including the pathophysiology of hypertension [42], atherosclerosis [43], depression [44, 45], and diabetes [46]. Obesity, in fact, is closely linked with inflammation as white adipose cells secrete inflammatory cytokines and adipokines that exacerbate systemic proinflammatory state, insulin resistance, and general morbidity [47–49]. The NF-kB family of proteins, RelA (p65), RelB, and c-Rel, and p100 and p105 which subsequently degrade to form p52 and p50, respectively, [50] are central to the regulation of inflammation [51]. To be able to present the full scope of NF-kB regulation and the transcription factor expression of its cognate genes could take a small book. However, here a selected understanding of the transcription factor's interactivity in the framework of the curcumin extract's pharmacology will be discussed.

Beyond even inflammation, NF-kB is a transcription factor that regulates networks which maintain cell health and survival and also plays a common and central role in disease pathology [52, 53]. The transcription factor system is a central mediator and conductor of the immune, inflammatory, oxidative, and stress responses [54–56]. It plays a central role in the mechanism of cancer, viral, and bacterial induction and survival [57, 58]. In fact, host responsiveness to viral infection such as with TNF*α* synthesis can, itself, activate NF-kB binding to DNA and transactivation to convert viruses like HIV-1 into their active forms [59].

The NF-kB family of proteins partakes in a complex expression of as many as 150 genes [55, 60–63] including key cytokines central to regulation of inflammatory and immune system activity: interleukin 2 (IL-2) [64, 65], interleukin 6 (IL-6) [66], interleukin 8 (IL-8) [67], interleukin 12 (IL-12) [68], TNF-*α* [69], and interleukin 1 (IL-1) [70]. Mutations of genes, such as NFkBIA, transcribing p65-p50 inhibitor protein, and I-kappa-B, are shown to be involved in the pathophysiology of autoimmune disorders where the transcription factor is uncontrollably freed to deliver constitutive activity [51, 71]. Otherwise, the NF-kB p65-p50 heterodimer is held inactive in the cytoplasm by the I-kappa-B repressor protein until I-kappa-B is phosphorylated by I-kappa-B kinase (IKK) at serines^32&36^ to set in motion its degradation by the 26S proteasome [72].

A rather simplistic model explaining the activation of the cytoplasmic p65-p50 heterodimer speaks to IKK activation by phosphorylation of its serine^176^ [73]. This IKK phosphorylation can be induced by a variety of upstream kinases as shown in Figure 2 and once phosphorylated it ultimately frees the heterodimer to facilitate p65-p50 translocation into the nucleus. The IKK complex is typically found as a heterotrimer in the cytoplasm as shown in Figure 2 or as a homodimer [74, 75] as seen in the alternate nonclassical transduction pathway of the same figure. These aggregated IKK isoforms cross-phosphorylate or crosstalk to facilitate their synergistic roles [76, 77]. The variable IKK configurations trigger different modalities by which free p65-p50 or p65-p52 transcription factors are, respectively, generated from different upstream receptors to make for varying translocation dynamics and transcriptional outcomes by the same family of transcription factors. It is a nonlinear, rather complex, system producing outcomes that can vary rheostatically and by gene target [23, 78].

Once uncoupled from its cytosolic repressor, p65-p50 can translocate into the nucleus [79] to engage in the transcription of genes with the kappa-B motif (GGG ACT TTC C) [58, 80]. This promoter nucleotide motif is essential for NF-kB docking. While posttranslational phosphorylation of p65 may be required for docking and expression of one gene, other posttranslational modifications of the transcription factor can prevent its docking on gene promoters [81–84] and transactivation despite the promoter regions of these genes containing the requisite kappa-B nucleotide motif. This phosphorylative coding helps shape p65-p50 transactivation selectivity after the transcription factor has translocated into the nucleus [83, 85] guiding it to highly specific genomic targets [86–88]. Despite the fact that the transcription factor potentially expresses as many as 150 different genes this phosphorylative coding, as will be further detailed, limits transactivation to the expression of genes which produce an appropriately measured behavioural response to the stimulus that started in motion the NF-kB activity [89–93].

Despite translocation, phosphorylation of the right p65 amino acid residues, and accessibility to a kappa-B equipped gene promotor, transcription by p65-p50 of the kappa-B TATA-less promoter also depends on the activation by phosphorylation of other nuclear transcription factors such as Sp1 [94, 95] and cAMP response element binding protein (CREB) [96]. The TATA box (consensus sequence TATAAAA) is located upstream of the start site of transcription and serves as a facilitator for transcription factor-promoter binding in higher eukaryotes [97]. The kappa-B promoter for which the p65-p50 transcription factor has affinity is TATA-less with the TATA region replaced by a “CG-rich” nucleotide sequence.

As a function of the TATA-less configuration, transcription by NF-kB depends on cotranscription factors that converge on the promoter to facilitate NF-kB docking and gene expression [98]. In the case of p65-p50, we see cotranscription factors Sp1 and CREB serving this purpose [94, 95]. Gene expression naturally also depends on histone phosphorylation and acetylation of chromatin to induce remodelling and accessibility by these converging cotranscriptional elements [96, 99–101]. Further to the old school understanding of IKK's cytosolic activation of p65-p50, once phosphorylated, IKK is more recently known to shuttle into the nucleus [99] and also partake in histone H3 phosphorylation which supports chromatin remodelling [102]. The shaping and regulation of NF-kB are complex and comprehensive to say the least but this complexity produces an opportunity for multiple points of regulation that we will see shortly. Curcumin is intimately interactive with and regulatory of this NF-kB signalling pathway at multiple points [23, 103, 104].  Figure 3 captures this interactive dynamic in schematic form.

## 3. MAPK Pathway Crosstalk with NF-kB Signalling Pathway

The MAPK pathway is also involved in this NF-kB shaping [105]. The MAPK pathway is an evolutionary ancient pathway like NF-kB's and is found in most species to control a vast array of cellular processes [106]. Curcumin is closely interactive with and regulatory of the MAPK pathway as well [107, 108]. This pathway interacts intimately with NF-kB [105, 109]. The MAPK pathway, like the NF-kB proteins and their pathways, is activated by inflammatory cytokines [110] and by environmental stressors to contribute to disease pathologies from tumorigenesis [111] to autoimmune diseases [112] and asthma [113, 114]. This central pathway plays a critical role in cell survival, apoptosis, and proliferation [115, 116]. NF-kB p65 (RelA) protein shaping by phosphorylation is complex [117–119] and begins in the cytoplasm, in part, by the MAPK (MEK/ERK) and continues into the nucleus all the way to transactivation and expression of the transcription factor's cognate genes [90, 105, 109, 120–123] as seen in Figures 2 and 3.

As previously discussed, phosphorylation of various p65 residues affords the NF-kB transcription factor the phosphorylative coding required to dock on some promoters for transactivation and not others once it has translocated. For example, phosphorylation of p65 serine^536^ is expected to be necessary for nuclear translocation but is also required for transcription of IL-6 [85]. However, lack of p65 serine^536^ phosphorylation abrogates p65 affinity for the IL-8 gene promotor [83]. Lack of serine^536^ phosphorylation does not, however, preclude the p65 heterodimer from docking and transcribing other cytokines, the genes of which are equipped with the kappa-B nucleotide motif.

Nuclear phosphorylation of the NF-kB p65 protein continues to shape the transcription factor's activity through nuclear kinases like the MAPK mitogen- and stress-activated kinase 1 (MSK1) [124, 125]. MSK1's nuclear activity is multifactorial and compounding in the context of NF-kB regulation. It phosphorylates and activates CREB [126], one of the p65-p50's cotranscription factors. In addition to MSK1, protein kinase A (PKA) [50, 125] coordinates a symphony of nuclear events that aggregate multiple cotranscription factors as well, including phosphorylation and activation of CREB [126] just like MSK1 does [105, 127]; activation of CBP/p300 and Sp1; and coordination of HDAC-1 to further contribute to chromatin remodelling [50]. These transcriptional elements all converge on and collaborate toward regulation of p65-p50 transactivation.

Both MAPK and NF-kB pathways are central to disease pathology and cell survival [115, 128–130]. Curcumin targets both these pathways at multiple points each as previously cited, portrayed schematically in Figure 2 [131–133], and discussed further in the pages to come.

## 4. Curcumin Helps Shape NF-kB p65-p50 Transactivation and Inflammation

Regulation of the NF-kB transcription factor is rather complex and the result of its modification can have profound implications based on the plethora of genes it transcribes [60–62]. To know that curcumin can inhibit p65-p50 activity at multiple phosphorylation sites sets the stage for an interesting investigative journey. However, to consider that the naturally occurring curcuminoid analogues comprising the curcumin extract have differing structural features that may be contributing different pharmacological characteristics leads to the cautious expectation that the true pharmacology of the curcumin extract has yet to be demystified.

The complexity of the subcellular events induced by curcuminoid preparations in various studies indicates that the curcuminoids are aimed at multiple biological targets where many of which guide NF-kB p65-p50 transactivation [23, 131, 134]. The effective inhibition of p65-p50 and inflammation by curcumin [135] heightens interest in the natural extract as a therapeutic agent [136]. However, curcumin pharmacology may be as complex and convoluted as the pleiotropic activity of the NF-kB family of transcription proteins. Curcumin is shown to inhibit IKK [23] as one component of the natural extract's pharmacology and as a result curcumin ultimately inhibits p65-p50 nuclear translocation [137, 138]. Curcumin inhibition of IKK is a classically accepted mechanism for the natural extract's anti-inflammatory activity [23, 138]. However, this cannot completely explain all the pharmacological outcomes shown in the literature with curcumin treatment [139]. Curcumin is shown to also inhibit PKC [140], which inhibits MAPK activity. Curcumin is also shown to inhibit Raf-1 [132, 133] which also reduces MEK signalling (MAPK) from yet another point in the MAPK pathway as seen in Figures 2 and 3. Inhibition of multiple cytosolic PKC isoforms [103, 140–142] plays a monumental role in MAPK pathway regulation [143, 144].

The symphony of curcuminoid activity on transduction through the MAPK pathway modulation and influences of NF-kB transactivation appears at first to be void of selectivity or strategy. However, once this plethora of activity is carefully mapped, a bigger picture begins to emerge. The pleiotropic influences by curcumin and its inherent curcuminoid analogues seem to be honing in, in a compounding manner, on inhibition of p65 activity. The extract's polypharmacology looks as though it is sharpening transactivation of p65-p50 synergistically through crosstalk by the MAPK pathway all the way into the nucleus. As mentioned, curcumin is also shown in the literature to inhibit PKA [145] delivering another level of compounding inhibitive activity on p65-p50 transactivation at the nuclear level.

Curcumin's pharmacology is complex. Curcumin is shown to inhibit transactivation of p65-p50 while still maintaining basal activity in healthy cells [146]. Curcumin is shown to induce apoptosis in mutated cells such as melanoma [146, 147] and to facilitate apoptosis by chemotherapies in drug-resistant cells improving drug efficacy [148]. This could be intimately related to its influence on NF-kB. Curcumin enhances caspase 8 activity [149]. However, at the same time, curcumin promotes malignant cell death and it preserves and protects healthy cells in the same environment from chemotoxicity [148]. Curcumin's pharmacology varies depending on cell type and receptor-ligand interaction triggering the cell response [150–152]. Different sources of cell stimulation such as LPS, TNF*α*, and TGF*β*, to name a few, which initiate transduction from different receptor sites create a differential p65 phosphorylation dynamic [129, 153].

The variable trigger points for inflammatory activity result in variations of the same p65 protein [85] that differ in their phosphorylation and heterodimer configurations; the outcome of which is multiple condition-specific responses from one transcription factor. Nevertheless, curcumin inhibits the different IKK isoforms involved in these different pathways and the different NF-kB heterodimer configurations that rise out of these different trigger points [23, 154, 155]. Still, despite all we know about the many mechanisms involved, the fundamental mechanism driving curcumin pharmacology is not fully understood since we are still discovering new targets and new interactions with conflicting results [156–160].

By modulating brain derived neurotrophic factor (BDNF) [161], curcumin performs as an antidepressant agent in a fashion similar to fluoxetine and imipramine [162]. Curcumin is shown to improve cardiac hypertrophy and heart failure in animal models [163, 164]. Curcumin can perform better than diclofenac sodium in the treatment of rheumatoid arthritis [6, 165]. In murine models of cystic fibrosis (CF), curcumin improves cystic fibrosis transmembrane conductance (CTFR) defects [166]. The extract helps improve muscle regeneration after injury [167]. Administration of curcumin improves cognitive function in Alzheimer's disease patients [168] and improves COPD-like airway inflammation [169]. Curcumin administration improves lipid metabolism to support healthier total cholesterol and HDL to LDL ratios associated with obesity [170–172].

However, as much as these and many other positive findings serve as a storyline for curcumin praise just as many studies demonstrate lack of efficacy with curcumin administration [173]; in depression models [174]; in CTFR (CF) defects [175]; and in rheumatoid arthritis and inflammatory bowel disease [176]. There is no doubt that curcumin can play a role in the management of inflammatory disease but in order for this to happen with greater reliability, the underlying mechanism must be better understood.

Curcumin has shown promise in the treatment of cancer [177], for instance, and in combination with paclitaxel can be effective in enhancing cytotoxicity in drug-resistant cancers [178–180]. Although the mechanism is understood to be centered on NF-kB inhibition by curcumin, the full story is still incomplete.

## 5. Curcumin-Based Therapy Challenges

A new perspective that involves two new viewpoints must be adopted in order to unveil some of the mystery still trapped within this natural extract.

Studying each of the curcuminoid analogues in isolation may help unravel some of the mystery surrounding this medicinal agent. Synthetic curcumin analogues, for example, can display unique pharmacological characteristics associated with structure [181, 182], structural differences that are rather miniscule. The naturally occurring curcuminoid analogues display similar structural characteristics, but as shown in Figure 1, their unique features may also contribute distinct pharmacological characteristics that are unique to each analogue. However, the naturally occurring curcuminoid analogues have not been studied expansively in this context in the past. A reevaluation of each of the curcuminoid analogues' pharmacology in isolation in the framework of NF-kB regulation may provide more insight into the full spectrum of curcumin activity and the source of the curcumin extract's polypharmacology.

*Curcuminoid nomenclature* also needs to be revisited. The whole 1 : 1 turmeric rhizome powder* (Curcuma longa)* will contain approximately 3–6% curcumin that comprises a mixture of the three naturally occurring curcuminoid analogues at concentrations that approximate 50–80% curcumin I, 10–20% curcumin II, and 0.5–2% curcumin III [31, 183–185]. Total curcumin content can be as high as 98% for a curcumin extract that retains these same curcuminoid analogue proportions inherently [139]. The term “curcumin” can refer to the principal curcuminoid, curcumin I (one), also called diferuloylmethane [186]. However, the term “curcumin” is confusingly also used in the literature and commercial applications, as will be shown, to describe the curcumin extract that contains all three curcuminoids (I, II, and III). To make matters related to consistency worse, the proportion of the naturally occurring curcuminoid analogues (I, II, and III) in curcumin extracts can vary from sample to sample contributing to a lack of standardization when comparing research executed with “curcumin.” This lack of nomenclature clarity must be more definitive.

Natural curcumin preparations that are standardized to a precise concentration, often as high as 95% curcumin, have within them these underlying variances that may be contributing to inconsistent outcomes. The assumption is that the curcuminoid analogues all display similar pharmacology. However, studies do point to the likelihood that the curcuminoids do not produce the same pharmacology on all targets. For example, bisdemethoxycurcumin (curcumin III aka BDMC) is shown to deliver cytotoxicity to inhibit growth of the K562 cell line and this inhibitory activity is significantly greater than that of curcumin (curcumin I aka diferuloylmethane) and demethoxycurcumin (curcumin II aka DMC) [187]. On the other end of the spectrum studies showed that curcumin I and demethoxycurcumin (curcumin II) have equally potent inhibitory activity on TPA induced tumorigenesis but bisdemethoxycurcumin (curcumin III) was less active [188]. The mechanisms are undefined and seemingly conflictive, nevertheless indicative of different activity by the different curcuminoid analogues.

The curcumin nomenclature does not help to make research initiatives clear. If we take an example of PKC inhibition by curcumin [189], Balasubramanyam et al. revealed that they acquired their curcumin for research from Sigma-Aldrich Co. with no more descriptive detail in the study. Another study demonstrating PKC inhibition by curcumin [103] where curcumin is “purchased from LKT Laboratories (>98%)” includes no more detail than this in the material description including no catalogue number for the item. Upon viewing the LKT catalogue, it is confirmed that this product appears to be almost exclusively curcumin I. Other researches showing that curcumin inhibits PKC [190, 191] simply state that curcumin is procured from Sigma and no more detail other than that.

Sigma, in another example, displays a 94% curcuminoid content curcumin (catalogue number C7727) but guarantees greater than 80% curcumin in the research standard. This product is obviously a curcumin extract, labelled “curcumin” but supplies multiple curcuminoids with the major curcuminoid analogue being curcumin I based on the molecular structure of the primary constituent shown in the product specification sheet. However, this product is more than just curcumin I; it also includes a nondescript array of other curcuminoids and as a result provides a variety of possible contributors to the pharmacology demonstrated in the study.

Inhibition of PKC by curcumin is shown in another study by Mahmmoud [192]. They too are using Sigma's curcumin (catalogue number C7727) which contains more than one curcuminoid analogue but the researchers refer to their inhibitor as “curcumin.” Catalogue number C7727 has only approximately 80% curcumin I and almost 20% other constituents. Inconsistent material specification may be playing a monumental role in the lack of reliability and repeatability of research and treatment outcomes. As we have demonstrated above, the curcuminoid analogues can display differing pharmacology and as submitted, standardization must be taken more seriously.

*Acknowledging the Pharmacological Contribution of the Curcumin Autooxidative By-Product*. High dose administration by oral route of some curcumin products results in limited to no serum curcumin in the subjects [24, 193], so why therapeutic results are still positive? Curcumin readily degrades in biological mediums and biological pH nonenzymatically to yield ferulic aldehyde, trans-6-(40-hydroxy-30-methoxyphenyl)-2,4-dioxo-5-hexenal, feruloyl methane, ferulic acid, and vanillin [194]. Although the studies have revealed conflictive evidence, some of these degradation by-products display significant pharmacological activity [195–197]. In addition, some, such as ferulic acid and vanillin, unlike the parent curcuminoids, display significant solubility and stability in biological mediums and at biological pH [198, 199]. If serum curcumin levels are regularly measured too low [24, 193] to account for pharmacological results after steady oral loading with curcumin, what is the source of the irrefutable results [200]? Are the nonenzymatic autooxidative degradation products responsible?

Curcuminoid degradation proceeds rapidly at pH above neutral, which is associated with biological mediums [198, 201]. Is curcumin or its degradation by-products responsible for the in vivo pharmacology at the site of activity? Are both classes,degradation and parent molecules, responsible? While curcumin is known to inhibit xanthine oxidase [202], ferulic acid, for example, a curcumin degradation by-product is also shown to inhibit xanthine oxidase reducing uric acid crystallization associated with gout [203–205]. Inhibition of xanthine oxidase can also reduce the intensity of many symptoms of disease including nonspecific symptoms associated with aging and chronic inflammation [206]. Xanthine oxidase escalates superoxide radical production, where overactivity simply produces additive biological stress [207]. Inhibition may play a functional role in disease management.

Ferulic acid administration can facilitate NO-mediated vasodilation [208]; pharmacology is also induced by curcumin administration [209]. Curcumin [210], just like ferulic acid [211], is shown to have significant antitumor activity. While we know that curcumin inhibits NF-kB [155, 212], ferulic acid is shown to do the same [213]. Curcumin [155, 212, 214–217], just like ferulic acid [197, 217–219], destabilizes preformed *β*-amyloid protein and inhibits stability of soluble oligomer and fibril aggregation. Vanillin, a curcumin degradation by-product, inhibits cyclooxygenase (COX), NF-kB, caspase-1 [220], and ischemia-induced hippocampal CA1 cell death [221]. Vanillin also protects neurons from oxidative stress [197].

**Figure 4:**
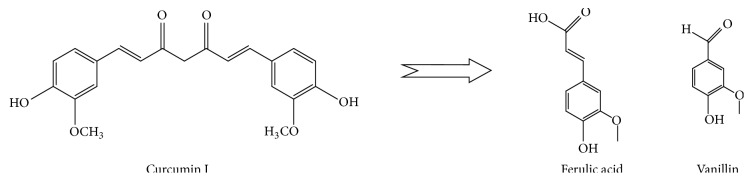
Diferuloylmethane's* (curcumin I)* nonenzymatic degradation yielding ferulic acid and vanillin as by-products.

**Figure 5:**
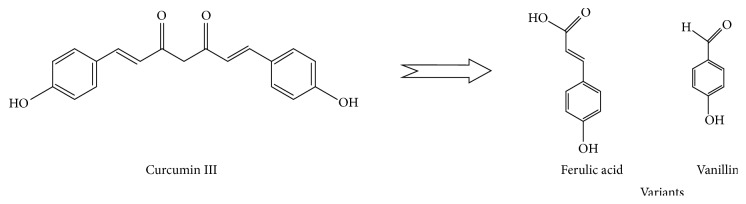
Bisdesmethoxycurcumin's (curcumin III) nonenzymatic degradation yield.

Since oral administration of some curcumin drugs is shown to result in low to zero serum curcumin even with prolonged high dose administration [18, 222], it leaves us with the degradation products as likely contributors, at least in part, to the broad polypharmacology attributed to curcumin. However, not all curcumin-related studies reflect the same curcumin bioavailability limitation so inevitably serum curcuminoids are playing a significant role as will be evidenced shortly. It must be considered that this autooxidative degradation of curcumin may not proceed as linearly as we would like to think.  Figure 4 displays the nonenzymatic degradation products of diferuloylmethane's (curcumin I) depicted again with ferulic acid and vanillin as by-products. However, curcumin III (bisdemethoxycurcumin) would produce degradation products with structure varying from those presented for curcumin I as seen in Figure 5.

In addition to the nonenzymatic autooxidative degradation of curcumin, curcumin is quickly metabolised enzymatically in an attempt by the body to neutralize and eliminate the natural agent. This enzymatic modulation starts in the intestinal lumen [223, 224] and subsequently, once absorbed into systemic circulation, yet another therapeutic barrier, the liver, imposes enzymatic neutralization. The metabolites resulting from this activity are quickly further subjected to glucuronation and sulfation to form curcumin glucuronide, curcumin sulfate, dihdrocurcumin glucuroside, tetrahydrocurcumin glucuronoside, and hexahydrocurcumin glucuronoside [223, 225–227]. This enzymatic neutralization contributes further to the elimination of serum curcumin and that low to no serum curcumin often associated with curcumin administration even at high oral doses. However, despite the apparent limitations associated with bioavailability, oxidative degradation and metabolic activity in vivo results persist albeit not reliably by all curcumin-containing products tested [216, 228–231, 231–233].

## 6. The Therapeutic Value of a Standardized Curcuminoid Treatment

Needless to say, curcumin's potential as a therapeutic agent is significant. However, there are some challenges that need to be overcome. Curcumin is known for its bioavailability limitations but this challenge may very well be overstated. Contrary to the stated bioavailability limitations, many studies such as that executed by Baum et al. applying regular curcumin extract in human clinical trials at daily doses of 1.0 grams showed significant serum curcumin (1100 + /−260 nM) within 1.5 hours [234]. In addition, the parent curcuminoids were also accompanied by significant serum levels of the nonenzymatic degradation by-products of curcumin. Poor curcumin/curcuminoid bioavailability is said to be caused by the phenolic compound's hydrophobic property and the consequential poor solubility in aqueous mediums [235].

Improving curcumin solubility in aqueous medium by complexing the curcuminoid with hydrophilic compounds like phosphatidylcholine improves solubility but is also purported to improve bioavailability [200]. However, these studies showing increased serum curcumin with reacted forms of curcumin that appear to have higher solubility [200] in aqueous solutions than curcumin alone might be missing another cause of improved serum curcumin, such as improved survival against hepatic enzyme modification [236]. In addition, improved solubility does not convey improved bioavailability. Even in studies that show low to no serum curcumin upon oral administration [24, 37, 193, 237] and excessive efflux [238] reported results from the therapies as previously mentioned are considered good, thus indicating from this standpoint that other factors, possibly even the autooxidative degradation by-products, are contributing pharmacology.

Regulation of the complex NF-kB transcription factor can play a significant role in disease management and improved cell survival [153, 239, 240]. Curcumin's biological activity is intimately interactive with NF-kB through multiple targets to downregulate the transcription factor and its involvement in disease pathology [23, 23, 155, 182]. Since inflammatory chemistry is central to all disease pathology in one form or another and is ultimately the target of disease treatment including diseases as difficult to treat as cancer [241–243], multiple sclerosis [244, 245], rheumatoid arthritis [245–247], ulcerative colitis [248, 249], Crohn's disease [250], and other autoimmune and autoinflammatory diseases [251, 252], NF-kB regulation is a highly targeted prospect in disease therapy [253].

Improved regulation of inflammatory markers by curcumin administration can lead to the potential improvement cognitive deficits [254], those aligned with Parkinson's disease pathology [255], Alzheimer's disease [168], and nonspecific oxidative brain damage [256, 257]. Obesity, as we have seen, is also closely linked with inflammation [47–49]. These age- and lifestyle-related diseases are North American epidemics today evolving to global pandemic status [258–260]. The right curcuminoid design could play a powerful therapeutic role in the treatment or prevention of many diseases including premature aging.

## 7. Future Direction for Curcumin Standardization in Research and Therapy

The future of curcumin is as bright as its pigmentation. However, in order for the pharmacology of this medicinal agent to be optimised, a better understanding of the distinct pharmacology of each naturally occurring curcuminoid analogue must be fully explored. The expansive polypharmacology is likely a function of the multiple targets successfully modulated by each curcuminoid analogue distinctly and the same targets they may successfully regulate to produce additive activity. In addition, it may be considered that the curcuminoid analogues successfully modulate distinct targets that act synergistically by crosstalk such as that seen between the MAPK and NF-kB pathways. In all, the total outcome of this polypharmacology may be one that plays out as a strategic corralling or shaping of NF-kB transactivation as described schematically in Figures 2 and 3.

Curcumin must be studied from this polypharmacological point of view in order to better understand the pharmacokinetics and pharmacology of each curcuminoid in isolation. This compartmentalized information will likely help us improve selective usage of curcumin-based strategies in research. It may even help improve the synergistic value hiding within the natural extract that may be unlocked by man-made drug designs that involve varying the curcuminoid analogue proportions within the curcumin-based treatment.

It is well established in some studies that bioavailability limitations are real with the hydrophobic curcumin but this is countered by studies that show otherwise. Commercially driven claims that the hydrophobic nature of the curcuminoid and its lack of solubility in aqueous mediums is the cause of curcumin bioavailability limitation is simply unfounded. Even with those demonstrating poor serum curcumin after heavy oral dosing, there is difficulty explaining the pharmacological results experienced by subjects receiving curcumin.

Serum curcumin levels are not found to be significant enough to explain therapeutic results in some studies [24, 37, 193, 223, 224] but as has been demonstrated in other researches, serum curcumin with unmodified curcumin extracts that are properly extracted can be significant and sufficient for efficacy [200]. However, despite the challenges, in vivo results can range from therapeutically great to mediocre from one curcumin-based product to another [216, 228–231, 231–233]. This indicates that other factors are playing pharmacological roles and also contributing activity. It is likely the internal proportion shifts of the curcuminoid analogues and the autooxidative by-products that must be considered in this pharmacological equation. The inconsistency and uncertainty are amongst the challenges that researchers need to overcome in order for the true value and full potential of this therapeutic agent to be extracted and put to good use.

Despite some attempts to improve curcumin bioavailability through liposomal and other forms of microencapsulation [261–263] and aerosol delivery [264], the effective hepatic degradation of the curcuminoids [223, 225–227] and that which can start in the lumen [265, 266] are shown to present yet another formidable therapeutic barrier contributing to serum insufficiency found with curcumin dosing. The conditions in which the curcumin-based therapies are studied also play a monumental role in the outcome seen in the literature. Serum proteins significantly improve curcumin survival [194] so once in the bloodstream a unique dynamic ensues. Factors such as pH, serum antioxidant status, and temperature all influence curcuminoid stability and autooxidative degradation [194, 267].

The status of the by-products of curcumin autooxidative degradation and their potential contribution to in vivo pharmacology must also be studied with greater sophistication such as starting with fundamental measurement of actual tissue distribution. Too much conflicting data has been presented in this context [38, 194, 268–270] and although the multiple viewpoints are great to see for meta-analysis, it must be considered that these conflicting positions could also be a function of the variable conditions being used to study the curcuminoids. Variable pH, temperature, serum protein, and other conditions, if even mildly varied, result in varying the degradation dynamic and outcome even at the analytical stage of serum samples after extraction.

Standardization of the curcumin extract to clearly define its constituent curcuminoid analogues at every juncture is crucial. Labelling and definitions of standards and consumer products must be made more universal. Curcumin extracts described on the label of a supplement source or natural therapeutic product, for example, may not be describing the same principal agent today from brand to brand due to these discrepancies in interpretation. Even within one brand, however, the variable proportion of curcuminoid analogues could also play a role in the inconsistency experienced from one lot number to the next using the existing regulatory standards. An extract displaying 95% curcumin purity, for instance, does not necessarily specify the curcuminoid proportions (I, II, and III). This lack of consistency extends to the peer-reviewed literature as well.

“Curcumin” is a descriptor often used to describe the curcumin extract which contains all three curcuminoids: curcumin I, curcumin II, and curcumin III. Confusingly, however, as described, “curcumin” is also used to describe curcumin I on a label. It must be established as a standard in commercial and research applications that a reference made to any one or three of the curcuminoids would be qualified by naming the specific curcuminoids. This nomenclature specificity must be standardized globally in order for label claims on consumable products, pharmacological agents, and research reports to be consistent in the health care field including peer-reviewed literature. Once this standard is set and adhered to we can begin to further define and better understand the expansive potential of this therapeutic agent with reliable and repeatable results.
